# Sex-Based Differences in Anthropometric Profiles and Physiological Responses to High-Intensity Strength–Endurance Exercise in Paramedic Students: A Cross-Sectional Study

**DOI:** 10.3390/jfmk11030359

**Published:** 2026-09-08

**Authors:** Robert Podstawski, Krzysztof Borysławski, Paweł Kukla, Jadwiga Snarska, Emilia Bałdyga, Attila Szabo, Alex MacQuarrie, Jayden R. Hunter, Paweł Jastrzębski

**Affiliations:** 1 Human Wellness Research Laboratory, Department of Physiotherapy, University of Warmia and Mazury in Olsztyn, Prawocheńskiego 13, 10-720 Olsztyn, Poland; amilus@wp.pl; 2 Faculty of Health Sciences, Angelus Silesius University of Applied Sciences in Wałbrzych, Zamkowa 4, 58-300 Wałbrzych, Poland; kboryslawski@ans.edu.pl; 3 Department of Emergency Medicine, Faculty of Health Sciences, Jagiellonian University in Cracow, Michałowskiego 12, 31-126 Kraków, Poland; pawel.kukla36@gmail.com; 4 Department of General Surgery, University of Warmia and Mazury in Olsztyn, Żołnierska 14C, 10-561 Olsztyn, Poland; jadwiga.snarska@uwm.edu.pl; 5 Faculty of Health and Sport Sciences, Széchenyi István University, Egyetem tér. 1, H-9026 Győr, Hungary; szabo.attila@sze.hu; 6 School of Health Sciences and Medicine, Bond University, Gold Coast, QLD 4215, Australia; smacquar@bond.edu.au; 7 Holsworth Biomedical Research Centre, La Trobe Rural Health School, La Trobe University, Bendigo, VIC 3550, Australia; j.hunter@latrobe.edu.au; 8 Department of Emergency Medicine, University of Warmia and Mazury in Olsztyn, Żołnierska 14C, 10-561 Olsztyn, Poland; pawel.jastrzebski@uwm.edu.pl

**Keywords:** cardiovascular reactivity, body composition, exercise tolerance, sex factors, physical activity

## Abstract

**Objectives:** This study evaluated sex differences in physical activity levels, anthropometric parameters, strength–endurance performance, and acute physiological responses to high-intensity strength–endurance exercise in emergency medical services students. **Methods:** Participants included 36 male and 23 female students aged 19–29 years. Body composition was assessed using bioelectrical impedance analysis. Physical activity was measured using the International Physical Activity Questionnaire, and strength–endurance performance was assessed with the 3-Minute Burpee Test (3-MBT). Blood pressure, heart rate, oxygen saturation, perceived exertion, and handgrip strength were recorded before and after the 3-MBT. **Results:** Men reported significantly higher PA levels than women (1963 vs. 1367 MET·min/week, *p* < 0.001). Women had a significantly higher percentage of body fat (31.0% vs. 19.4%, *p* < 0.001). Men completed more 3-MBT cycles than women (44 vs. 33 cycles/3 min, *p* < 0.001), whereas the between-sex difference in mean heart rate was not significant after Benjamini–Hochberg correction (170.2 vs. 162.2 bpm; adjusted *p* = 0.075). Systolic blood pressure was higher in men before and after the test (*p* < 0.001 and *p* = 0.004, respectively) and increased significantly in both sexes (*p* < 0.001). Oxygen saturation and handgrip strength decreased significantly after the 3-MBT in both groups (*p* < 0.001). **Conclusions:** Men demonstrated greater strength–endurance performance than women, although both sexes were classified as having “poor” performance according to established 3-MBT normative values. Men reported higher physical activity, whereas women had a higher body fat percentage. These findings demonstrate sex-based differences in physical activity, body composition, and strength–endurance performance among paramedic students.

## 1. Introduction

Paramedics perform one of the most cognitively and physically demanding roles in healthcare, delivering time-critical care in complex, unpredictable and often hazardous environments. They must simultaneously assess, treat, and transport patients while managing high cognitive load, heavy equipment, and awkward patient-handling tasks, making adequate physical capacity fundamental to safe and effective practice [1]. Paramedics experience high rates of occupational injury, with sprains, strains, and body-motion–related injuries consistently identified as leading causes [2,3,4]. Evidence indicates that female paramedics experience a higher proportional injury rate, and that injury risk is influenced by both physical demands and workforce demographics [5]. These injuries often arise during manual handling, repeated lifting and carrying, and stretcher operations—tasks that require strength, muscular endurance, and appropriate body composition [5]. Manual handling remains a major mechanism of musculoskeletal injury despite ongoing training initiatives.

Rising service demand, expanding scopes of practice, and aging workforces further increase the need for sustained physical performance over long careers [6]. Physical tasks such as lifting and carrying patients, negotiating stairs or uneven terrain, and performing prolonged cardiopulmonary resuscitation impose considerable cardiometabolic and neuromuscular demands [7,8]. The ability to perform such occupational tasks depends largely on strength–endurance capacity, which reflects the integrated function of the neuromuscular, cardiovascular, and metabolic systems. Sustained high-intensity muscular performance requires effective motor unit recruitment, adequate muscle strength, appropriate muscle fiber characteristics, oxidative capacity, and fatigue resistance [9,10]. Body composition and habitual physical activity also contribute to strength–endurance performance, as greater fat-free mass and regular physical activity are associated with improved muscular function and exercise capacity [11,12,13]. Furthermore, sex-related differences in muscular performance are well documented and are mainly attributed to differences in skeletal muscle mass, muscle cross-sectional area, hormonal profile, and neuromuscular function. Although men generally demonstrate greater absolute strength, women may show greater fatigue resistance during selected submaximal tasks due to differences in muscle morphology, perfusion, and metabolic responses [13,14,15]. These physiological differences may influence occupational performance and injury susceptibility during physically demanding emergency medical tasks.

Studies of paramedics and other tactical responders demonstrate that high-intensity functional tasks can elicit substantial cardiovascular strain, rapid fatigue, and measurable performance decrements under stress [8]. Inadequate physical capacity may therefore elevate injury risk, impair movement quality, and alter physiological stress responses in ways that affect decision-making and technical skill. This may in turn lead to adverse patient outcomes [16,17]. Objective evaluation of physical activity, body composition, and acute physiological responses to high-intensity efforts is essential for determining whether paramedics—and those preparing to enter the profession—possess the functional readiness required for frontline clinical duties. However, existing research predominantly focuses on qualified paramedics [7], with comparatively little attention to the physical preparedness of students approaching entry to practice. Some evidence suggests that physiological stress reactivity may be heightened during paramedic training, underscoring the need to understand how candidates respond to intense workloads [18].

Although sex-based differences in strength, body composition, and physical fitness have been extensively investigated [19], previous research has predominantly focused on athletes, military personnel, and general populations [14,15]. Studies have demonstrated consistent sex-related differences in muscular strength, fatigue resistance, and physiological responses to exercise; however, the magnitude of these differences depends on the type of task, exercise intensity, and characteristics of the studied population [13,20]. Comparatively little attention has been given to paramedic students, despite the high physical demands associated with their future professional responsibilities. Existing research has mainly evaluated qualified paramedics and tactical responders, whereas the physical preparedness and acute physiological responses of individuals entering this profession remain insufficiently explored [7,8]. Moreover, limited evidence is available regarding the combined assessment of anthropometric characteristics, habitual physical activity, strength–endurance performance, and cardiovascular responses during high-intensity functional exercise in paramedic students. Therefore, this study aimed to address this research gap.

The primary aim was to evaluate sex-based differences in strength–endurance performance and acute physiological responses to a high-intensity 3-Minute Burpee Test among male and female paramedic students. The secondary aims were to assess sex-based differences in physical activity levels and anthropometric characteristics. We hypothesized that male and female paramedic students would differ in anthropometric characteristics, habitual physical activity levels, strength–endurance performance during the 3-MBT, and selected acute physiological responses to the test.

## 2. Materials and Methods

### 2.1. Study Design

This was a cross-sectional observational study. The study protocol involved a single assessment session, during which anthropometric measurements, physical activity levels, and physiological responses to a high-intensity exercise test were evaluated.

### 2.2. Participants

The study was conducted from 1 April to 15 April 2025 (summer semester) in the Human Research Wellness Laboratory at the University of Warmia and Mazury in Olsztyn (UWM). All 59 students enrolled in the same year of the emergency medical services program were invited to participate, and all 59 students (100%) participated in the study, including 36 men and 23 women, aged 19–29 years (mean ± SD: men: 21.25 ± 1.87 years; women: 21.52 ± 1.34 years). Participants were recruited from the university’s emergency medical services program and met the inclusion criteria of being currently enrolled in the program, medically cleared for high-intensity exercise, and free from injury at the time of testing. Thus, all students in the respective academic cohort were included in the study, with no participants selected from outside this cohort. As all participants were enrolled in the same academic year and followed the same curriculum, the study population was relatively homogeneous with respect to educational background.

### 2.3. Ethical Statement

The research was conducted in accordance with the guidelines and policies of the Health Science Council and the Declaration of Helsinki. The University of Warmia and Mazury Ethics Committee approved the study (approval no. 39/2011, issued on 24 November 2011). Prior to participation, all students received written and verbal explanations of the study’s purpose, procedures, and potential risks. Participants were informed that they could withdraw at any time without penalty. The protocol included detailed demonstrations of all measurement procedures and motor fitness testing techniques, which participants practiced during familiarization sessions immediately before data collection. All participants provided voluntary, written informed consent.

### 2.4. Assessment of Participants’ Physical Activity Levels

Participants’ physical activity (PA) levels were assessed using the Polish short version of the International Physical Activity Questionnaire (IPAQ) [21]. Prior to data collection, participants reported the average weekly duration (in minutes) of PA per bout (minimum of 10 min). Energy expenditure associated with the declared weekly PA levels was expressed in metabolic equivalent of task (MET) minutes per week [22]. A MET represents the ratio of the metabolic rate during activity to the resting metabolic rate, where 1 MET corresponds to an estimated oxygen consumption of 3.5 mL·kg^−1^·min^−1^. Based on the declared frequency, intensity, and duration of their reported PA (“how often, how much, how long”), participants were categorized into one of three PA levels: low PA (<600 MET·min/week), moderate PA (600–1500 MET·min/week), or high PA (≥1500 MET·min/week).

### 2.5. Procedures, Data Collection, and Equipment

The study was conducted in the Human Research Wellness Laboratory of the University of Warmia and Mazury in Olsztyn, Poland. Participants were instructed to avoid strenuous physical activity for 24 h before testing and to maintain their usual hydration status on the day of the study. Participants were also instructed to avoid alcohol, caffeine, and other stimulants before the assessment, as these factors may influence acute cardiovascular responses to exercise. Anthropometric measurements were performed in the morning (08:00–09:00 a.m.) after an overnight fast under standardized laboratory conditions (ambient temperature 22–24 °C, relative humidity 40–60%). Participants were assessed barefoot and wearing light clothing in accordance with the Martin anthropometric procedures. Upon arrival at the laboratory, anthropometric measurements, including body composition assessment by bioelectrical impedance analysis, were performed before any additional fluid intake. After completing these measurements, participants were allowed to drink approximately 0.5 L of water to ensure adequate hydration before subsequent physiological measurements and motor fitness tests, which commenced between 11:00 a.m. and 1:00 p.m. Because the entire testing session lasted several hours and included physical exercise, participants were also advised to consume at least 1 L of water throughout the day to maintain adequate hydration. During physiological measurements and motor fitness testing, participants wore standard sports clothing and athletic footwear. Anthropometric assessments were completed before the motor fitness tests to eliminate the acute effects of exercise on body composition measurements.

### 2.6. Anthropometric Measurements and Body Composition Analysis

Body height was measured to the nearest 1 mm using a calibrated InLab S50 stadiometer (InBody Co., Seoul, Republic of Korea) in accordance with standard anthropometric guidelines. Body mass (to the nearest 0.1 kg), body mass index (BMI), and body composition parameters, including body fat percentage (BFP) and skeletal muscle mass (SMM), were assessed using a dual-frequency bioelectrical impedance analyzer (InBody 270, Biospace Co., Ltd., Seoul, Republic of Korea). The InBody 270 employs direct segmental multi-frequency bioelectrical impedance analysis using two frequencies (20 and 100 kHz) and an eight-point tactile electrode system with tetrapolar electrode configuration to assess body composition. Previous research has demonstrated that BIA is a valid method for estimating body composition and provides results comparable to dual-energy X-ray absorptiometry (DXA) [23,24]. Previous studies have also confirmed the reliability and validity of the InBody 270 [25,26].

### 2.7. The 3-Minute Burpee Test

Strength-endurance abilities were assessed using the 3-Minute Burpee Test (3-MBT) as described by Podstawski et al. [27]. The 3-Minute Burpee Test (3-MBT) was used to assess strength-endurance capacity because it engages major muscle groups and the cardiovascular system simultaneously, reflecting the physical demands of emergency medical work [28]. The test evaluates the ability to perform repeated high-intensity whole-body movements, which is essential for tasks such as patient handling, cardiopulmonary resuscitation, and prolonged emergency operations. An additional advantage of the 3-MBT is its simplicity, standardized administration, and the lack of requirement for specialized equipment [29]. During the regular practical classes in the five weeks preceding the experimental study, participants completed one familiarization 3-MBT session per week (five sessions in total) to ensure proper technique and reliable performance during the formal testing [28]. Before the formal test, participants were instructed in how to perform the 3-MBT correctly and given time to practice the full movement sequence. Participants performed the 3-MBT technique as previously described by Podstawski et al. [30]. The 10-min active warm-up consisted of 3 min of light jogging at a self-selected intensity, 3 min of dynamic mobility exercises involving major muscle groups (including the upper and lower limbs and trunk), 2 min of specific movement preparation involving squats, push-ups, and burpee technique practice, and 2 min of dynamic stretching. The warm-up protocol was standardized for all participants and was performed before each 3-MBT attempt, as an appropriate warm-up may enhance physical performance and prepare individuals for high-intensity exercise [31].

### 2.8. Assessment of Physiological Parameters

During each 3-MBT test, participants’ physiological parameters, including heart rate (HR; minimum, average, and peak values), energy expenditure (kcal), and exercise intensity based on HR responses, were assessed indirectly using a heart rate monitoring system consisting of a Polar H10 chest strap sensor and Polar V800 receiver (Polar Electro, Kempele, Finland), which have been widely used in studies of this type [32]. The heart rate sensor was positioned on the participant’s chest according to the manufacturer’s recommendations, and a researcher operated the monitoring system. This procedure enabled standardized recording of physiological responses immediately before, during, and after each 3-MBT. Before testing, each Polar V800 device was configured with participants’ basic characteristics, including sex, body mass, height, and year of birth. To assess exercise intensity, a reference maximal heart rate (HRmax) of 162 bpm was applied uniformly to all participants. This value served as the reference HRmax for the Polar system in determining the corresponding heart rate zones. Applying the same HRmax and corresponding HR zones to all participants ensured a standardized and consistent classification of exercise intensity across the study sample. Exercise intensity during the 3-MBT was determined from HR responses using the Polar Flow application, which automatically calculated the duration spent in each heart rate zone. The HR zones applied in the present study were as follows: Zone 1 (<90 bpm), Zone 2 (90–108 bpm), Zone 3 (109–125 bpm), Zone 4 (126–144 bpm), Zone 5 (145–162 bpm), and Zone 6 (>162 bpm). This classification was consistent with the methodology applied in our previous investigations using the same assessment protocol [33].

Blood pressure was measured with an Omron M6 Comfort blood pressure meter (Omron, Kyoto, Japan), and peripheral oxygen saturation (SpO_2_) was measured with an ORO-OXIMETER finger pulse oximeter (Oromed Szymanek Sp. K., Poznań, Poland). Blood pressure was assessed in a seated position using the left arm and an appropriately sized cuff. Measurements were performed immediately before exercise and immediately after exercise, with the post-exercise measurement initiated within 30 s of exercise cessation. A single measurement was performed at each time point to minimize the time elapsed between exercise cessation and post-exercise assessment, as repeated measurements could have resulted in a substantial decline in blood pressure during the measurement period. If the device displayed an error, the measurement was repeated. This protocol was used to assess changes in blood pressure and SpO_2_ associated with maximal exercise. Given the immediate post-exercise timing, the potential influence of acute post-exercise physiological changes and measurement-related error should be considered when interpreting the blood pressure results.

### 2.9. Assessment of Handgrip Strength

Bilateral handgrip strength was measured using a standardized digital dynamometer (MG4800, Charder Electronic Co., Ltd., Taichung, Taiwan). The strain-gauge-based device converts mechanical force into an electrical signal, which is displayed on a digital screen. Measurements were performed in a seated position according to the recommendations of the American Society of Hand Therapists (ASHT), with participants seated upright, feet flat on the floor, shoulders adducted and neutrally rotated, elbows flexed at 90°, and the forearm and wrist in a neutral position. Hand dominance was determined based on participants’ self-report, with the dominant hand defined as the hand preferentially used during daily activities. Three trials were performed for each hand, with a standardized 1-min rest interval between attempts. The highest value obtained for each hand was recorded. Handgrip strength was assessed immediately before and 60–90 s after completion of the 3-MBT using the same standardized procedure for all participants.

### 2.10. Rating of Perceived Exertion (RPE)

Participants’ rating of perceived exertion (RPE) was assessed immediately after completion of the 3-Minute Burpee Test using the Borg 6–20 Rating of Perceived Exertion Scale. Before testing, all participants received standardized verbal instructions on how to use the scale and were asked to rate their overall perception of physical effort experienced during the test. The English version of the Borg 6–20 scale was used because it is an internationally recognized and extensively validated instrument for assessing perceived exercise intensity, with good validity and reliability across different populations [34].

### 2.11. Statistical Analysis

Descriptive statistics (mean, standard deviation, and range) were calculated to characterize the study sample. The Shapiro–Wilk test was used to assess the normality of the distribution of all analysed variables, and skewness coefficients were examined to further assess distributional properties. Homogeneity of variances was assessed using Levene’s test. Differences between males and females were assessed using the independent-samples Student’s *t*-test for variables showing a normal distribution and no significant difference in variances between the groups. For variables that did not meet these assumptions, the Mann–Whitney U test was applied; these variables are indicated in italics in the tables. Effect sizes were calculated as Cohen’s d for parametric comparisons or as the rank effect size r (r = Z/√N) for Mann–Whitney U tests. Within-group differences between measurements obtained before and after exercise were assessed using the paired-samples Student’s *t*-test. To account for multiple comparisons, *p*-values were adjusted using the Benjamini–Hochberg false discovery rate (FDR) procedure, applied separately to each set of comparisons presented in the respective tables. We set the significance level at α = 0.05. No missing data were recorded, and all participants completed the full assessment protocol; therefore, no data imputation was required. All statistical analyses were performed using Statistica version 13.0.

## 3. Results

Table 1 presents the descriptive characteristics of physical activity levels and body composition parameters for men and women. Men demonstrated a significantly higher level of physical activity than women (*p* < 0.001), with a mean difference of 596 MET·min/week. Based on IPAQ guidelines, men were classified as reporting high PA (≥1500 MET·min/week), while women were classified as reporting moderate PA (600–1500 MET·min/week). Men had significantly greater body height, body mass, fat-free mass (FFM), and skeletal muscle mass (SMM) than women (all *p* < 0.001), consistent with established population trends. In contrast, parameters reflecting adiposity (BMI, WHR) did not differ significantly between sexes (*p* > 0.05), whereas BFP, BFM, and VFL were significantly higher in women (31.0% vs. 19.4%, *p* < 0.001; 22.41 vs. 17.23 kg, *p* = 0.013; and 9.74 vs. 7.08, *p* = 0.019, respectively). Mean BFP values suggested that men (19.4%) fell within normal ranges, whereas women (31.0%) showed elevated adiposity according to established classification criteria [35]. Among women, 12 of 23 participants (52.2%) had BFP values of ≥30%.

Despite the absence of significant differences in BMI, the mean BMI in men (25.8 kg/m^2^) was at the lower end of the overweight range, whereas the mean BMI in women (24.8 kg/m^2^) was at the upper limit of the normal range. Notably, BFM values differed significantly between sexes, with women showing a modestly higher mean BFM (+5.2 kg). The mean WHR in men (0.89) was slightly below the WHO threshold associated with increased cardiometabolic risk (≥0.90), whereas the mean WHR in women (0.90) exceeded the corresponding threshold for women (≥0.85), suggesting relatively greater central fat accumulation. The InBody Score, a manufacturer-derived indicator reflecting overall body composition status, did not differ significantly between sexes after correction for multiple comparisons (*p* = 0.044; adjusted *p* = 0.072), with mean scores of 77.9 in men and 73.0 in women.

Table 2 presents sex-related differences in physiological parameters recorded during the 3-MBT, values obtained during the 6-min recovery period, and pre- and post-exercise measurements of systolic blood pressure (SBP), diastolic blood pressure (DBP), heart rate (HR), oxygen saturation (% SpO_2_), and handgrip strength. After completing the 3-MBT, participants reported very high perceived exertion, with mean RPE scores of 18.8 in men and 19.3 in women, indicating near-maximal effort. During the 3-MBT, men completed almost 11 more cycles than women (*p* < 0.001). Men also exhibited a higher mean HR than women (+8 bpm), although this difference did not remain statistically significant after Benjamini–Hochberg correction (adjusted *p* = 0.075). Women spent significantly more time in Zone 3 (*p* = 0.013; adjusted *p* = 0.049), whereas the between-sex differences in time spent in Zones 4 and 5 did not remain statistically significant after correction (adjusted *p* = 0.090 and 0.130, respectively). During the 6-min recovery period, men also spent more time in intensity Zone 6, although the difference was not statistically significant (*p* = 0.225). No significant sex differences were observed in minimum or maximum HR values. Energy expenditure during the test was higher in men (≈60 kcal) than in women (≈50 kcal) (*p* < 0.001). Men showed significantly higher SBP values than women both before and after the 3-MBT (*p* < 0.001 and *p* = 0.004, respectively), while DBP values did not differ significantly between sexes at either time point. Similarly, we detected no sex differences in HR or %SpO_2_ before or after the test. Men exhibited significantly greater handgrip strength in both the dominant and non-dominant hands, both before and after the motor test (*p* < 0.001).

Table 2 also reports pre- and post-exercise values for SBP, DBP, HR, %SpO_2_, and handgrip strength in both sexes. SBP increased significantly in men and women following the 3-MBT (both *p* < 0.001), with large effect sizes (Cohen’s d = 1.050 for men; 1.378 for women). In contrast, DBP decreased significantly only in women (*p* = 0.007; Cohen’s d = 0.519), while no significant change was observed in men. Both sexes experienced significant reductions in oxygen saturation, with large effect sizes (men: *p* < 0.001, Cohen’s d = 1.313; women: *p* < 0.001, Cohen’s d = 1.653). Similarly, dominant handgrip strength decreased significantly in men (*p* < 0.001; Cohen’s d = 0.623) and women (*p* < 0.001; Cohen’s d = 0.473), and non-dominant handgrip strength also declined significantly in men (*p* < 0.001; Cohen’s d = 0.645) and women (*p* < 0.001; Cohen’s d = 0.659).

## 4. Discussion

The aim of this study was to assess sex differences in physical activity, anthropometric parameters, strength-endurance capacity, and acute physiological responses to high-intensity effort amongst emergency medical services students. Men reported high levels of physical activity, whereas women reported moderate levels, reflecting the well-established trend of greater habitual activity in male university students [36,37].

Interestingly, despite marked sex differences in height and body mass favoring men, the present study found significantly higher absolute body fat mass (BFM) in women. This finding, together with the higher percentage of body fat observed in women, indicates that the greater overall body size of men did not translate into greater absolute adiposity in this sample. These results highlight the importance of evaluating body composition rather than body size alone when interpreting physical readiness and health-related risk. Reports in the literature are not uniform in this respect, and the magnitude of the sex-related difference in absolute fat mass appears to depend on the characteristics of the population examined and on the adjustments applied. Clinical studies using DXA have shown that when comparing the body composition of men and women with overweight/obesity, total fat mass can be similar between sexes after adjusting for age and BMI, even though fat distribution patterns differ substantially between women and men [38]. Similarly, Caudwell et al. [39] found that during a 12-week exercise program, men and women lost different amounts of total body mass, yet reductions in fat mass and body fat percentage were comparable, with no statistically significant differences between sexes (*p* > 0.05). In contrast, and in line with the present findings, Youssef et al. [40] reported that female students exhibited higher total fat mass and percent body fat, while male students demonstrated higher fat-free mass, although differences in other health-related indicators were inconsistent or not statistically significant.

In the present sample, the estimated visceral fat level (VFL) was likewise significantly higher in women than in men (9.74 vs. 7.08; *p* = 0.019). This observation deserves comment, because studies using imaging-based reference methods generally indicate greater visceral adiposity in men at a comparable BMI [38]. In the present cohort, however, the higher VFL in women was accompanied by a higher BFP and a higher absolute BFM, and by a mean WHR that exceeded the sex-specific threshold associated with increased cardiometabolic risk (0.90 vs. a threshold of ≥0.85), whereas the mean WHR in men remained below the corresponding threshold for men (≥0.90). Comparable sex-related differences in body composition and fat distribution have been described in other samples of university students [41,42]. This result should nevertheless be interpreted with caution, because the VFL returned by the InBody 270 is an ordinal index derived from impedance and anthropometric input rather than a direct measurement of visceral adipose tissue, and it was not verified against computed tomography, magnetic resonance imaging, or DXA in the present study.

Men significantly outperformed women in strength-endurance abilities measured by the 3-MBT. According to international standards developed by Podstawski et al. [30], both sexes achieved a “poor” performance classification, completing 43.8 cycles/3 min in men and 33.1 cycles/3 min in women, values notably lower than those reported for European university students (men: 56.7 cycles/3 min; women: 48.8 cycles/3 min). However, these classifications were derived from general university student populations rather than occupation-specific standards for paramedics and should therefore not be interpreted as direct indicators of professional readiness. In a 2012 study, Polish female university students achieved an average of 39.9 cycles/3 min [43]. These earlier findings also demonstrated that each 1% increase in BMI corresponded to a 0.93% reduction in repetitions completed during the 3-MBT, underscoring the sensitivity of this test to excess body mass. Performance varied markedly by BMI category: underweight female students achieved the highest scores (47 cycles/3 min), followed by those in the normal BMI range (41 cycles/3 min), whereas overweight female students performed substantially worse (29.1 cycles/3 min). In a 2016 study, female participants completed an average of 48.6 cycles/3 min, with heart rate reaching 182 bpm at the end of the test and decreasing to 151 bpm within 90 s [27]. Previous studies have reported negative associations between body mass/BMI and 3-MBT performance [44]. However, the observed sex-related differences in 3-MBT performance may not be explained solely by differences in body size and adiposity. Neuromuscular factors, including sex-related differences in motor unit recruitment, muscle activation strategies, contractile properties, and resistance to fatigue, may also influence the ability to sustain repeated high-intensity whole-body movements. These mechanisms may be particularly relevant during the 3-MBT, which requires continuous activation [13].

The findings of the present study are consistent with those reported by Podstawski et al. [44], who similarly observed that physical activity levels, fitness profiles, and body composition indicators tend to be more favorable in male than in female university students. In that study, strength–endurance performance was approximately 23.75% higher in men than in women (47.2 vs. 37.2 cycles/3 min), mirroring the significant sex differences observed in the current investigation. Likewise, men in the 2019 study spent significantly more time in the maximal effort zone during the 3-MBT (*p* = 0.047), whereas in the present sample the corresponding difference in Zone 5 (145–162 bpm) did not remain statistically significant after correction for multiple comparisons (adjusted *p* = 0.130), and no significant between-sex difference was observed in Zone 6 (>162 bpm; *p* = 0.715). However, while men reached higher maximal HR values than women in the 2019 cohort (185.1 bpm vs. 173.3 bpm; *p* < 0.001), the present study did not identify sex-based differences in maximal HR, suggesting that HR responses during maximal calisthenic effort may vary across cohorts despite consistent performance disparities.

Interestingly, despite the greater external work performed by men (44 vs. 33 cycles), no statistically significant sex differences were observed in mean HR after correction for multiple comparisons, and no sex differences were observed in SpO_2_ responses during the test. This may indicate that both groups achieved a comparable level of physiological strain relative to their individual capacities, whereas the greater number of cycles completed by men likely reflected sex-related differences in muscular strength and mechanical performance [45]. The maintenance of SpO_2_ values suggests that both groups preserved adequate oxygenation during high-intensity effort, indicating sufficient oxygen transport capacity without evidence of exercise-induced desaturation [46]. Although HRmax is generally considered relatively independent of sex and fitness level, previous studies have reported small sex-related differences in maximal heart rate responses [47]. In the present study, HR_max_ did not differ significantly between men and women, suggesting that maximal heart rate responses were broadly comparable despite the difference in 3-MBT performance.

The Borg Rating of Perceived Exertion (RPE) scale confirmed that the 3-MBT elicited an extreme, near-maximal effort, consistent with previous descriptions of the upper range of the scale [48]. High-intensity strength–endurance exercise is known to increase systolic blood pressure (SBP), whereas diastolic blood pressure (DBP) typically remains stable [49,50], and the present findings partially support this pattern. Following the 3-MBT, SBP increased significantly in both sexes (*p* < 0.001), while DBP decreased significantly in women (*p* = 0.007) and remained unchanged in men. The observed reduction in DBP among women may reflect sex-specific differences in vascular responses to acute exercise, including variations in peripheral vascular resistance and autonomic regulation. Post-exercise reductions in DBP have been associated with transient decreases in vascular resistance and altered cardiovascular regulation following strenuous exercise [51]. However, post-exercise blood pressure measurements obtained using oscillometric devices may also be affected by transient physiological changes and measurement-related factors; therefore, this finding should be interpreted with caution.

The 3-MBT also produced a significant reduction in SpO_2_ in both men and women, reflecting the physiological challenge imposed by very high-intensity exercise. Under extreme metabolic and ventilatory demand, oxygen delivery may become insufficient relative to muscular oxygen utilization, resulting in a transient decline in arterial oxygen saturation. This response reflects factors such as increased muscular oxygen extraction, rightward shifts in the hemoglobin dissociation curve, and ventilatory limitations during intense exertion [52]. Such reductions in SpO_2_ are well-documented in both sexes, regardless of exercise modality, and are characteristic of severe anaerobic and strength–endurance efforts [53]. Mucci et al. [54] further observed that some individuals experience oxygen desaturation greater than 4% during high-intensity interval training, illustrating the potential magnitude of exercise-induced hypoxemia under high metabolic load.

The present findings indicate that paramedic candidates have relatively low motor abilities, with both men and women achieving “poor” 3-MBT scores compared with typical university students. This performance gap is concerning given the high physical demands of frontline paramedic work, which requires repeated whole-body effort in challenging environments. Rather than reducing structured activity in the curriculum, these results support reshaping existing exercise components into specialized activation programs that explicitly target job-relevant tasks (e.g., lifting, carrying, floor-to-stand transitions, and brief high-intensity efforts). Such targeted conditioning may help address observed deficits in motor abilities, enhance physical readiness for practice, and potentially reduce the risk of future injury as students transition into the workforce. The observed reduction in handgrip strength following the 3-MBT is consistent with fatigue from high-intensity exercise. Although the forearm muscles are not the primary movers during burpees, their role in stabilization and weight-bearing contributes to localized fatigue, while the overall physiological demand produces systemic fatigue. Reductions in force production post-intense exercise are well documented [55,56]. Handgrip strength has been shown to indicate overall neuromuscular function and may reflect changes in neuromuscular performance following demanding physical exercise [57]. In the present study, the observed reduction in handgrip strength after the 3-MBT may indicate acute neuromuscular fatigue; however, this interpretation should be considered with caution, as handgrip strength was not compared with independent markers of fatigue.

Women’s substantially lower strength–endurance performance in this study has important implications for paramedic practice, where high-force, short-burst tasks such as patient handling, stretcher maneuvering, and prolonged CPR impose considerable mechanical and metabolic demands on responders. Paramedics experience disproportionately high rates of musculoskeletal injury, and female paramedics have been shown to sustain proportionally higher injury rates than men [5], suggesting that lower baseline strength–endurance capacity may elevate strain during physically demanding tasks. Additionally, national physical-demands analyses demonstrate that paramedics are routinely exposed to high whole-body loading, awkward postures, and time-critical exertion [8], all of which amplify the relevance of adequate strength–endurance capacity. High-intensity functional simulations also show large increases in heart rate, metabolic load, and fatigue during task-specific exertion [58], underscoring the need for sufficient strength–endurance to maintain both performance and safety in operational environments.

Despite these performance differences, lower strength–endurance in women should not be viewed as an inherent limitation but rather as an indication that structured conditioning, ergonomic supports, and system-level interventions are essential. Evidence shows that many paramedics, regardless of sex, enter training or practice with suboptimal fitness relative to occupational demands [59], emphasizing that this is a workforce-wide challenge rather than a sex-specific deficiency. Furthermore, exposure to real-world stressors, including demanding clinical placements and operational loads, can augment cardiovascular strain during high-intensity efforts [18], resulting in a higher strain-to-capacity ratio among individuals with lower starting fitness. Finally, high-quality manual-handling training that incorporates biomechanics, physical conditioning, realistic simulations, and behavioral-change principles is more effective than traditional technique-only instruction [60], highlighting the importance of integrated strategies that ensure operational readiness while supporting equity across sexes. Future research should establish the criterion validity of the 3-Minute Burpee Test by examining its relationship with simulated and real-world paramedic tasks, such as patient handling, stretcher loading, prolonged cardiopulmonary resuscitation, and equipment carriage, to determine whether test performance predicts occupation-specific physical capability.

### Strengths and Limitations

A major strength of this study is that it evaluated the effects of a 3-min high-intensity endurance–strength exercise on fundamental physiological parameters, including heart rate (HR), blood pressure (BP), oxygen saturation (SpO_2_), and handgrip strength of both hands. Additionally, the study provides a comprehensive characterization of the anthropometric and motor profiles (endurance–strength performance) of future paramedics, accounting for sex-related differences. These findings provide valuable information on the physical characteristics and exercise responses of this population.

The study is limited by its small sample size, although it included the entire accessible cohort from one paramedic science program, providing a homogeneous and representative sample of the target population but limiting generalizability and statistical power. Body composition was assessed by BIA under standardized laboratory conditions, but its indirect nature and reliance on proprietary algorithms require cautious interpretation. Although all BIA measurements were taken in the morning after an overnight fast and before any additional fluid intake, hydration status was not verified objectively (for example, by urine specific gravity or plasma osmolality); because bioelectrical impedance is sensitive to total body water, residual differences in hydration between participants may have affected the body composition estimates, including BFP, BFM, and VFL. Exercise intensity was classified using uniform heart-rate zones rather than individualized %HRmax or %HRR zones. Sleep was not objectively assessed, testing was conducted within a limited daytime window rather than at a fixed time, and menstrual cycle phase and hormonal contraceptive use were not controlled; all of these factors may have influenced physiological responses and performance, although the study was designed to reflect real-world paramedic conditions.

## 5. Conclusions

The present study demonstrated that male paramedic students exhibited significantly higher strength–endurance performance than their female counterparts; however, both sexes were classified as having poor strength–endurance capacity according to established 3-MBT normative values. Men also reported higher levels of physical activity, whereas women had a greater percentage of body fat, factors that may have contributed to the observed differences in exercise performance. The 3-MBT elicited marked acute physiological responses in both sexes, including increased heart rate and systolic blood pressure, reduced oxygen saturation, and decreased handgrip strength, confirming its utility as a practical field test for evaluating physiological strain and fatigue following high-intensity whole-body exercise. Considering the substantial physical demands of emergency medical services, the generally low strength–endurance capacity observed in this cohort suggests that further investigation of the relationship between physical fitness and performance in occupation-specific paramedic tasks is warranted. Therefore, assessment and development of strength–endurance capacity should be considered important components of paramedic education to improve physical preparedness and support safe performance of occupation-specific tasks. The findings provide baseline information on strength–endurance capacity and acute physiological responses in paramedic students and support further research examining whether these measures are associated with performance in occupation-specific tasks.

## Figures and Tables

**Table 1:** Descriptive statistics—general characteristics of male and female participants.

Parameters	Male (n = 36)				Female (n = 23)						Difference			
	Mean	SD	Min	Max	As	Mean	SD	Min	Max	As	t (Z)	*p*	*p* (q) *	Effect Size
Age [Years]	21.25	1.87	19	29	2.931	21.52	1.34	20	25	1.487	*−1.56*	*0.118*	*0.177*	*0.203*
PA [MET·min/week]	1963.42	559.04	870	3020	−0.214	1367.39	441.41	780	2280	0.474	4.32	<0.001	<0.001	1.153
Body height [cm]	181.71	6.31	169.9	194.7	0.096	168.43	5.84	155.4	179.2	−0.438	8.16	<0.001	<0.001	2.166
Body mass [kg]	85.19	18.27	64.3	131.7	0.896	69.95	11.91	44.7	103.2	0.768	3.54	<0.001	0.001	0.941
BFM [kg]	17.23	11.30	4.4	49.6	1.329	22.41	9.96	7.7	49.3	1.131	*−2.49*	*0.013*	*0.025*	*0.324*
FFM [kg]	67.41	9.67	49.6	93.7	0.685	47.54	5.50	33.4	59.8	−0.348	8.95	<0.001	<0.001	2.390
SMM [kg]	38.47	5.84	27.5	54.4	0.665	26.19	3.33	17.6	33.7	−0.332	9.16	<0.001	<0.001	2.446
BMI [kg/m^2^]	25.76	5.20	18.7	41.2	1.175	24.76	4.85	17.4	37.4	1.325	*0.55*	*0.581*	*0.697*	*0.072*
BFP [%]	19.38	9.50	4.9	49.4	1.174	31.04	8.75	11.5	47.8	0.092	*−4.38*	*<0.001*	*<0.001*	*0.570*
InBody Score	77.86	11.82	34	104	−1.269	73.04	8.13	56	88	−0.307	*2.01*	*0.044*	*0.072*	*0.262*
Target weight [kg]	79.47	9.59	67.5	102.2	0.809	62.26	4.79	52.1	70.0	−0.462	*6.33*	*<0.001*	*<0.001*	*0.824*
Weight control [kg]	−5.72	12.65	−53.3	11.6	−2.056	−7.69	10.19	−33.2	9.2	−0.856	*1.37*	*0.162*	*0.208*	*0.177*
BFM control [kg]	−6.67	11.97	−53.3	4.4	−2.322	−8.53	9.29	−33.3	1.1	−1.267	*1.39*	*0.162*	*0.208*	*0.178*
FFM control [kg]	0.95	1.98	0.00	7.8	2.497	0.84	2.21	0.00	8.1	2.270	*0.18*	*0.858*	*0.858*	*0.023*
BMR [kcal]	1826.03	209.03	1441	2394	0.683	1396.74	118.89	1091	1661	−0.353	8.95	<0.001	<0.001	2.389
WHR	0.89	0.09	0.74	1.08	0.434	0.90	0.07	0.80	1.02	0.310	−0.19	0.715	0.775	0.051
VFL	7.08	5.73	1	26	1.561	9.74	5.22	2	22	0.931	*−2.35*	*0.019*	*0.034*	*0.305*
Obesity degree	117.03	23.66	85	187	1.170	117.91	23.16	83	178	1.327	*−0.34*	*0.732*	*0.775*	*0.044*

Notes: As—skewness, SD—standard deviation, PA—physical activity, BFM—body fat mass, FFM—fat-free mass, BFP—body fat percentage, SMM—skeletal muscle mass, BMI—body mass index, BMR—basal metabolic rate, WHR—waist-to-hip ratio, VFL—visceral fat level; effect size: d-Cohen’s for t-Student test or the rank effect size for the Mann–Whitney U test; italics—statistics for the Mann–Whitney U test. * The *p*-value after Benjamini–Hochberg correction (FDR = 0.05).

**Table 2:** Sex-Based Differences in Physiological Responses and Recovery Parameters During and After the 3-Minute Burpee Test.

Parameters	Male (n = 36)					Female (n = 23)					Difference			
	Mean	SD	Min	Max	As	Mean	SD	Min	Max	As	t (Z)	*p*	*p* (q) *	Effect Size
Physiological parameters for the 3-MBT														
3-MBT [Number of cycles]	43.86	8.47	27	69	0.925	33.09	7.61	16	44	−0.519	4.95	<0.001	<0.001	1.323
Perceived exertion	18.83	1.16	16	20	−0.823	19.30	0.76	18	20	−0.601	−1.72	0.091	0.238	0.460
HR min [bpm]	108.33	13.78	85	140	0.884	109.96	16.96	76	135	−0.716	−0.40	0.688	0.780	0.108
HR avg [bpm]	170.19	11.76	135	191	−0.385	162.22	14.14	136	191	−0.110	2.35	0.022	0.075	0.626
HR max [bpm]	188.08	8.72	169	202	−0.624	189.44	7.38	171	201	−0.419	−0.62	0.541	0.759	0.164
Energy expenditure [kcal]	59.75	8.80	42	72	−0.336	49.83	10.23	32	70	0.213	3.96	<0.001	<0.001	1.058
Zone 1 (<90 bpm) [s]	2.9	4.7	0	22	2.377	3.7	7.5	0	26	2.302	*−0.45*	*0.625*	*0.758*	*0.059*
Zone 2 (90–108 bpm) [s]	8.1	7.6	0	33	1.215	10.1	10.2	0	39	1.005	*−0.54*	*0.592*	*0.758*	*0.070*
Zone 3 (109–125 bpm) [s]	11.6	8.8	0	41	1.331	21.2	16.5	0	58	0.999	*−2.48*	*0.013*	*0.049*	*0.323*
Zone 4 (126–144 bpm) [s]	26.7	20.1	4	79	1.427	40.9	28.9	3	119	0.712	*−1.83*	*0.029*	*0.090*	*0.238*
Zone 5 (145–162 bpm) [s]	62.9	40.7	1	134	0.072	42.8	30.4	5	101	0.517	*1.72*	*0.046*	*0.130*	*0.224*
Zone 6 (>162 bpm) [s]	67.7	59.5	0	169	0.436	61.2	56.7	0	166	0.505	*0.37*	*0.715*	*0.784*	*0.048*
Physiological parameters for the 6-min recovery period														
HR min [bpm]	110.67	10.97	89	128	−0.249	114.09	8.62	98	128	0.143	−1.27	0.211	0.403	0.338
HR avg [bpm]	125.39	11.75	102	143	−0.563	128.87	8.20	117	143	0.333	−1.24	0.220	0.403	0.331
HR max [bpm]	185.18	8.51	168	201	−0.613	184.30	9.05	166	198	−0.644	0.37	0.707	0.260	0.099
Energy expenditure [kcal]	66.75	12.46	41	83	−0.549	66.74	10.15	50	83	−0.106	0.01	0.997	0.997	0.000
Zone 1 (<90 bpm) [s]	14.1	47.8	0	247	4.005	7.4	24.5	0	85	3.140	*0.51*	*0.608*	*0.759*	*0.066*
Zone 2 (90–108 bpm) [s]	81.1	85.7	0	264	0.914	100.5	68.8	0	203	−0.145	*−1.13*	*0.257*	*0.437*	*0.147*
Zone 3 (109–125 bpm) [s]	130.7	81.9	15	297	0.382	150.3	62.3	20	268	−0.191	*−0.87*	*0.380*	*0.615*	*0.113*
Zone 4 (126–144 bpm) [s]	87.7	68.5	6	248	1.264	63.9	28.6	34	163	2.066	*0.75*	*0.456*	*0.674*	*0.098*
Zone 5 (145–162 bpm) [s]	31.6	22.6	0	87	0.461	30.1	22.5	10	92	1.641	*0.25*	*0.804*	*0.854*	*0.033*
Zone 6 (>162 bpm) [s]	14.8	15.5	0	61	1.382	7.8	6.9	0	26	1.298	*1.21*	*0.225*	*0.403*	*0.158*
Differences in BP, HR, SpO_2_, and hand-grip strength before and after the 3-MBT														
SBP before [mmHg]	134.61	11.54	111	155	−0.131	123.52	10.90	100	148	−0.037	3.68	0.001	0.003	0.982
SBP after [mmHg]	153.92	23.32	112	199	0.006	136.78	12.36	115	156	−0.388	*2.87*	*0.004*	*0.017*	*0.374*
differences	t = 6.39, *p* < 0.001					t= 6.60, *p* < 0.001								
DBP before [mmHg]	74.70	8.64	52	91	−0.184	75.74	9.65	57	96	0.252	−0.43	0.667	0.780	0.116
DBP after [mmHg]	70.89	12.46	50	99	0.618	70.65	9.95	53	86	−0.250	0.08	0.939	0.968	0.020
differences	t = −1.87, *p* = 0.057					t = −2.97, *p* = 0.007								
HR before [bpm]	81.14	13.59	64	122	1.459	86.00	12.81	58	115	0.493	−1.37	0.176	0.399	0.366
HR after [bpm]	142.44	12.65	119	180	0.490	144.17	8.25	122	160	−0.334	−0.58	0.563	0.759	0.155
differences	t = 22.60, *p* < 0.001					t = 22.68, *p* < 0.001								
SpO_2_ before [%]	97.78	0.96	95	99	−0.552	98.09	0.85	96	99	−0.664	−1.26	0.212	0.403	0.337
SpO_2_ after [%]	96.25	1.34	93	98	−1.167	96.52	1.04	93	98	−1.529	−0.83	0.412	0.637	0.221
differences	t = −6.53, *p* < 0.001					t = −11.33, *p* < 0.001								
Dominant hand before [kg]	55.68	6.79	44.3	72.5	0.357	36.24	6.25	23.4	48.8	0.254	11.06	<0.001	<0.001	2.951
Dominant hand after [kg]	51.40	6.95	37.5	64.3	0.005	33.55	5.05	21.2	43.6	−0.226	10.64	<0.001	<0.001	2.840
differences	t = −7.72, *p* < 0.001					t = −4.82, *p* < 0.001								
Non-dominant hand before [kg]	51.47	6.27	42.3	61.9	0.235	33.40	5.22	22.9	42.8	−0.286	11.50	<0.001	<0.001	3.069
Non-dominant hand after [kg]	47.77	5.15	38.5	58.6	0.314	30.16	4.59	20.1	37.0	−0.635	13.35	<0.001	<0.001	3.563
differences	t = −7.39, *p* < 0.001					t = −5.16, *p* < 0.001								

Notes: 3-MBT—3-Minute Burpee Test; HR—heart rate; Zone 1–Zone 6—exercise intensity zones; SBP—systolic blood pressure; DBP—diastolic blood pressure; SpO_2_—blood oxygen saturation (percentage of hemoglobin saturated with oxygen); effect size: d-Cohen’s for T-Student test or the rank effect size for the Mann–Whitney U test, italics—statistics for the Mann–Whitney U test. * The *p*-value after Benjamini–Hochberg correction (FDR = 0.05).

## Data Availability

The access to Excel data generated during this study has been restricted by the Ethics Committee of the University of Warmia and Mazury in Olsztyn to protect the participants’ privacy. Researchers who meet the criteria for access to confidential data can submit a data request by email to podstawskirobert@gmail.com.

## Abbreviations

The following abbreviations are used in this manuscript:

PA	Physical activity
3-MBT	3-Minute Burpee Test
HR	Heart rate
SBP	Systolic blood pressure
DBP	Diastolic blood pressure
SpO_2_	blood oxygen saturation
As	skewness
BFM	body fat mass,
FFM	fat-free mass
BFP	body fat percentage
SMM	skeletal muscle mass
BMI	body mass index
BMR	basal metabolic rate
WHR	waist-to-hip ratio
VFL	visceral fat level
RPE	Rating of Perceived Exertion
IPAQ	International Physical Activity Questionnaire
MET	Metabolic Equivalent of Task
BIA	Bioelectrical Impedance Analysis
ASHT	American Society of Hand Therapists
FDR	False Discovery Rate
HRR	Heart Rate Reserve
BP	Blood pressure
CPR	Cardiopulmonary resuscitation
DXA	Dual-energy X-ray absorptiometry
